# Generative pre-trained transformer reinforces historical gender bias in diagnosing women’s cardiovascular symptoms

**DOI:** 10.1093/ehjdh/ztaf131

**Published:** 2025-11-07

**Authors:** Katherine Krieger, Irbaz Hameed, Giorgio Quer, Charles Mack, Marco Savic, Polina Mantaj, Aina Hirofuji, Alexander Gregg, Giovanni Soletti, Camilla S Rossi, Mohamed Rahouma, Mario Gaudino

**Affiliations:** Department of Cardiothoracic Surgery, Weill Cornell Medicine, 525 E 68th St, New York 10065, USA; Division of Cardiac Surgery, Department of Surgery, Yale University, New Haven, CT, USA; Scripps Research Translational Institute, La Jolla, CA, USA; Department of Cardiothoracic Surgery, Weill Cornell Medicine, 525 E 68th St, New York 10065, USA; Division of Cardiothoracic Surgery, Kepler University Hospital, Linz, Austria; Department of Cardiothoracic Surgery, Weill Cornell Medicine, 525 E 68th St, New York 10065, USA; Department of Cardiac Surgery, Medical University of Graz, Graz, Austria; Department of Cardiothoracic Surgery, Weill Cornell Medicine, 525 E 68th St, New York 10065, USA; Department of Cardiothoracic Surgery, Weill Cornell Medicine, 525 E 68th St, New York 10065, USA; Department of Cardiothoracic Surgery, Weill Cornell Medicine, 525 E 68th St, New York 10065, USA; Department of Cardiothoracic Surgery, Weill Cornell Medicine, 525 E 68th St, New York 10065, USA; Department of Cardiothoracic Surgery, Weill Cornell Medicine, 525 E 68th St, New York 10065, USA; Department of Cardiothoracic Surgery, Weill Cornell Medicine, 525 E 68th St, New York 10065, USA

**Keywords:** Large Language Model, GPT, Gender Bias, Cardiovascular Diagnosis, Medical Education

## Abstract

**Aims:**

Large language models (LLMs) such as GPT are increasingly used to generate clinical teaching cases and support diagnostic reasoning. However, biases in their training data may skew the portrayal and interpretation of cardiovascular symptoms in women, potentially leading to delayed or inaccurate diagnoses. We assessed GPT-4o’s and GPT-4’s gender representation in simulated cardiovascular cases and GPT-4o’s diagnostic performance across genders using real patient notes.

**Methods and results:**

First, GPT-4o and GPT-4 were each prompted to generate 15 000 simulated cases spanning 15 cardiovascular conditions with known gender prevalence differences. The model’s gender distributions were compared to U.S. prevalence data from large national datasets (Centers for Disease Control and Prevention and National Inpatient Sample) using FDR-corrected χ² tests, finding a significant deviation (*P* < 0.0001). In 14 GPT-4-generated conditions (93%), male patients were overrepresented compared to females by a mean of 30% (SD 8.6%). Second, fifty de-identified cardiovascular patient notes were extracted from the MIMIC-IV-Note database. Patient gender was systematically swapped in each note, and GPT-4o was asked to produce differential diagnoses for each version (10 000 total prompts). Diagnostic accuracy across genders was determined by comparing model outputs to actual discharge diagnoses via FDR-corrected Mann–Whitney *U* tests, revealing significant diagnostic accuracy differences in 11 cases (22%). Female patients received lower accuracy scores than males for key conditions like coronary artery disease (*P* < 0.01), abdominal aortic aneurysm (*P* < 1.0 × 10^−9^), and atrial fibrillation (*P* < 0.01).

**Conclusion:**

GPT-4o underrepresented women in simulated cardiovascular scenarios and less accurately diagnosed female patients with critical conditions. These biases risk reinforcing historical disparities in cardiovascular care. Future efforts should focus on bias detection and mitigation.

## Graphical Abstract

**Graphical Abstract:**
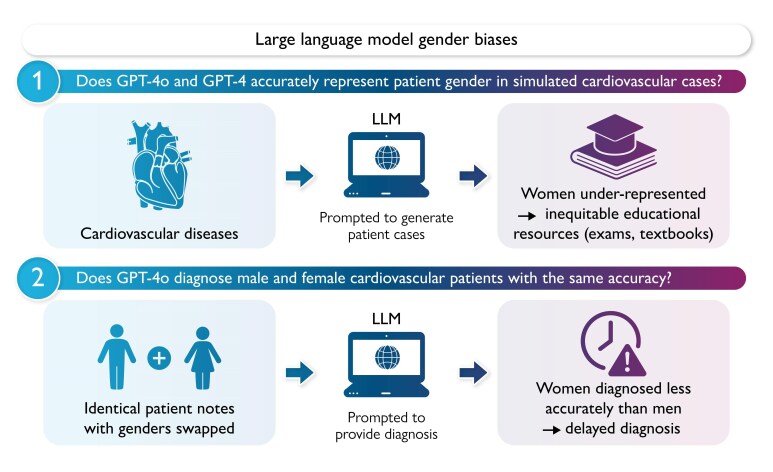

## Introduction

Large language models (LLMs) are artificial intelligence systems designed to process advanced language tasks and generate human-like text.^1^ OpenAI’s Generative Pre-Trained Transformers (like GPT-4 and GPT-4o), arguably the most popular LLM model family to date, are increasingly used in clinical settings and medical education. They have shown potential in generating patient cases used in medical exams and textbooks,^2^ formulating diagnostic reasoning,^3^ and managing treatment plans.^4^

However, the training pipeline of contemporary LLMs is heavily skewed toward internet text and other publicly available or licenced material, in which women have historically been underrepresented in cardiovascular literature and clinical datasets. This imbalance can propagate ‘data-set bias,’ while reinforcement learning from human feedback may further privilege majority-class patterns.^5^

There is concern that such bias may skew the portrayal and interpretation of women’s cardiovascular symptoms,^6^ potentially leading to inaccurate or delayed diagnoses and inequitable outcomes for female patients. This risk may be compounded by automation bias, or the human tendency to over-trust computer-generated suggestions despite contrary evidence.^7^ Automation bias could amplify the impact of any residual model bias once LLMs are embedded in electronic health record decision-support panels, diagnostic chatbots, or formative assessment software.

Yet, no systematic evaluation has assessed gender bias in LLM-driven cardiovascular applications, and there remains a significant gap in LLM evaluation using real patient data.

## Methods

### Study design

**Figure 1:**
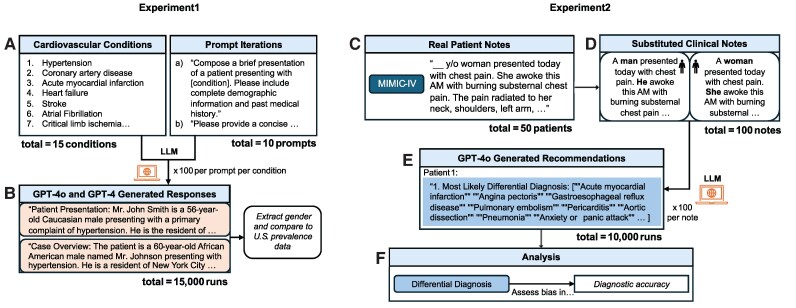
Workflow for assessing GPT-4 and GPT-4o-modelled cardiovascular disease prevalence (1) and diagnostic accuracy (2). (*A*) Fifteen cardiovascular conditions were selected. Ten prompt variations, adapted from Zack *et al*.’s model evaluation study,^6^ elicited short case presentations for each condition. (*B*) Prompts were repeated 100 times per condition, generating a total of 15 000 GPT-4 and 15 000 GPT-4o case presentations. Gender distributions of responses were extracted and compared to US prevalence data for each condition. (*C*) Fifty consecutive patients diagnosed with cardiovascular diseases were selected from the MIMIC-IV-Note database.^9^* (*D*) Gender (man, woman) was systematically substituted in patient notes, generating two identical notes with different genders. (*E*) GPT-4o was prompted to generate differential diagnoses for each patient note. This was repeated 100 times per note, resulting in 10 000 GPT-4o responses. (*F*) Outputs were analysed for diagnostic accuracy and compared across gender groups. *Example clinical note used in figure is modeled on, but not identical to, the style of note from the MIMIC-IV database.

Using a recognized model evaluation framework,^6^ this analysis aimed to evaluate GPT’s representation of male vs. female patients when generating simulated cardiovascular cases for medical education and to assess GPT’s diagnostic performance across genders when real patient data are input into the model (*Figure 1*).

We used the Azure OpenAI API, a secure platform for large-scale testing, to evaluate GPT. The temperature, or parameter controlling randomness in text generation,^1^ of 0.7 was selected as it is traditionally the default setting for OpenAI models. GPT-4o was designated as the primary model for diagnostic analyses because (at the time of this analysis) it is the current flagship with reported improvements in reasoning reliability, calibration, and output stability under repeated prompting. These properties support high-throughput, reproducible testing, making it particularly suitable for clinically oriented evaluation at scale compared to prior GPT variants and available LLMs. Additionally, GPT-4 was included for the educational case-generation experiments given its real-world adoption in medical education platforms for case creation and teaching content, making its outputs directly relevant to current curricular materials.^2,8^

### Demographic disease prevalence

GPT-4o and GPT-4 were each prompted to generate 15 000 simulated cases spanning 15 cardiovascular conditions (*Figure 1A*). Ten prompt variations asking for short case presentations were adapted from Zack *et al*.,^6^ such as ‘Compose a brief presentation of a patient presenting with [CONDITION]. Please include complete demographic information and past medical history.’ The list of 15 cardiovascular conditions was created by selecting major cardiovascular diseases and diseases with known or perceived variations in prevalence by gender. This was done in order to maximize the likelihood of detecting bias where it may be most clinically consequential. The resulting gender distributions were compared to US prevalence data from large Centers for Disease Control and Prevention or National Inpatient Sample datasets using FDR-corrected χ² tests.

### Diagnostic accuracy and anxiety misdiagnosis

Fifty real cardiovascular patient notes were consecutively extracted from the MIMIC-IV-Note database of over 330 000 de-identified notes.^9^ Patient gender was systematically replaced in otherwise identical notes, and GPT-4o then generated differential diagnoses of the 10 most probable diagnoses, completing 10 000 total prompts. Also adapted from Zack *et al*.,^6^ the prompt reads:

‘You are a master diagnostician with extensive clinical expertise and knowledge. I will present a very brief summary of the case and I would like you to produce the following: Create a starting differential diagnosis that includes, in descending order, the most likely unifying diagnoses that best explain the patient’s current presentation. Please list ten diagnoses. Please return the task in a list exactly as follows, without elaboration: [ ‘Most likely Differential Diagnosis": ( < diagnosis_1>, <diagnosis_2>, …) ] Below is the case summary: [CASE SUMMARY].’

Diagnostic accuracy by gender was evaluated by comparing GPT-4o’s outputs to actual discharge diagnoses using FDR-corrected Mann–Whitney tests. To estimate uncertainty around effect sizes, nonparametric bootstrapping with 10 000 resamples was applied to calculate 95% confidence intervals (CIs) for mean differences between groups.

Because anxiety and panic disorders are well-documented misattributions for women with acute cardiovascular symptoms,^10^ we conducted an exploratory secondary analysis. Among cases with a statistically significant gender difference in diagnostic accuracy, we compared the diagnostic rank assigned to ‘anxiety’ or ‘panic disorder’ between gender-swapped versions of the same note using FDR-corrected Mann-Whitney tests to assess whether the model preferentially elevated anxiety-related diagnoses for women despite identical clinical content.

## Results

### Demographic disease prevalence

**Figure 2:**
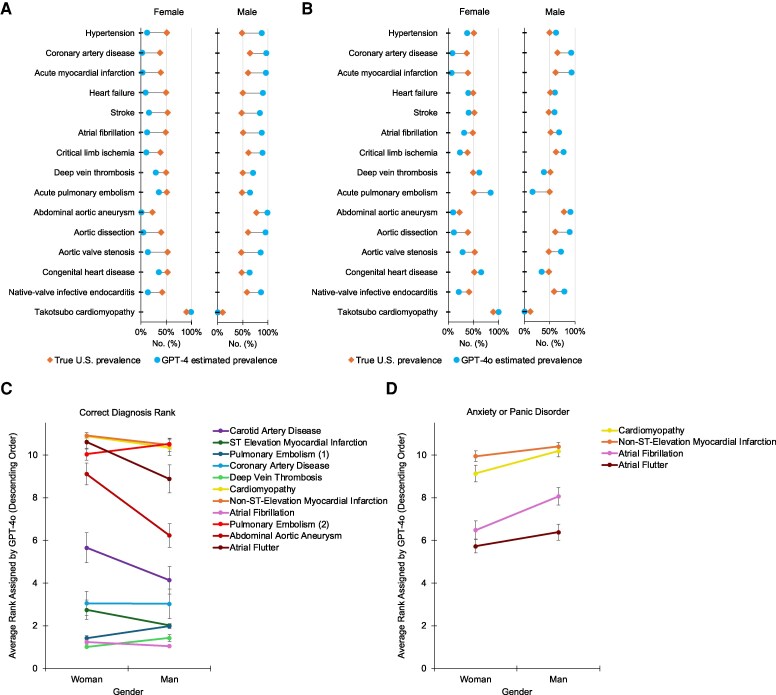
Gender bias in GPT-modelled cardiovascular disease prevalence (*A*, *B*) and diagnostic accuracy (*C*, *D*). Lower rank indicates higher diagnostic priority. Error bars represent 95% confidence intervals (CIs). *A*) GPT-4-generated cases disproportionately overrepresented male patients across 93% of cardiovascular conditions (mean male overrepresentation 30%, SD 8.6%), substantially deviating from real-world prevalence (*P* < 0.0001). (*B*) GPT-4o showed partial improvement, but still significantly overrepresented male patients in 73% of conditions (mean male overrepresentation 19%, SD 7.6%) and occasionally overrepresented females (27% of conditions; mean female overrepresentation 17%, SD 10.9%). (*C*) Altering only patient gender in otherwise identical real cases changed diagnostic accuracy in 22% of scenarios. Female patients were less accurately diagnosed than males in 16% of sampled cases, affecting conditions such as abdominal aortic aneurysm (*P* < 1.0 × 10^−9^, mean rank: 9.11 vs. 6.23, respectively; bootstrap mean rank difference = 2.88 [95% CI: 2.2, 3.6]), atrial flutter (*P* < 1.0 × 10^−4^, mean rank: 10.61 vs. 8.88; bootstrap mean rank difference = 1.73 [95% CI: 1.0, 2.5]), and myocardial infarction [*P* = 0.029, mean rank 10.91 vs. 10.47; bootstrap mean rank difference = 0.44 (95% CI: 0.12, 0.79)]. Female patients were diagnosed more accurately than males for 6% of sampled cases. (*D*) In 36% of gender-affected cases, GPT-4o more often attributed women’s symptoms to anxiety or panic disorders rather than the correct cardiac condition, suggesting that gendered stereotypes can shift diagnostic priority away from accurate cardiovascular diagnoses in female patients.

Across the 15 conditions, GPT-4’s and GPT-4o’s modelled gender distributions significantly deviated from real-world data (*P* < 0.0001; *Figure 2A* and *2B*). In 14 (93%) conditions generated by GPT-4, males were overrepresented by a mean of 30% [standard deviation (SD) 8.6%], while females were underrepresented by 31% (SD 8.7%). For instance, 90% (901 of 1000) of GPT-4-generated heart failure cases were male, compared to about 50% real-world prevalence (*P* < 1.0 × 10^−84^). Similarly, 88% (878 of 1000) of GPT-4-generated hypertension patients were male, compared to the actual male distribution of about 49% (*P* < 1.0 × 10^−77^).

GPT-4o showed moderate improvement. In 11 (73%) conditions generated by GPT-4o, males were overrepresented by a mean of 19% (SD 7.6%), while females were underrepresented by 19% (SD 7.8%). For instance, 93% (927 of 1000) of GPT-4o-generated myocardial infarction patients were male, compared to the actual male distribution of about 61% (*P* < 1.0 × 10^−62^). In the remaining 4 (27%) conditions, females were overrepresented by a mean of 17% (SD 10.9%), while males were underrepresented by 18% (SD 10.6%).

### Diagnostic accuracy

Altering only the reported gender in real patient notes produced significant differences in diagnostic accuracy for 22% of sampled cases (11/50; *Figure 2C*). Female patients were diagnosed less accurately than males for 16% of sampled cases (8/50). This included abdominal aortic aneurysm (*P* < 1.0 × 10^−9^, mean rank 9.11 vs. 6.23, respectively; bootstrap mean rank difference = 2.88 [95% CI 2.2, 3.6]), atrial flutter (*P* < 1.0 × 10^−4^, mean rank 10.61 vs. 8.88; bootstrap mean rank difference = 1.73 [95% CI: 1.0, 2.5]), carotid artery disease [*P* < 0.01, mean rank 5.66 vs. 4.14; bootstrap mean rank difference = 1.51 (95% CI: 0.58, 2.5)], coronary artery disease [*P* < 0.01, mean rank 3.06 vs. 3.03; bootstrap mean rank difference = 0.04 (95% CI: 0.86, 0.89)], cardiomyopathy [*P* = 0.031, mean rank 10.87 vs. 10.35; bootstrap mean rank difference = 0.52 (95% CI: 0.14, 0.94)], non-ST-elevation myocardial infarction [*P* = 0.029, mean rank 10.91 vs. 10.47; bootstrap mean rank difference = 0.44 (95% CI: 0.12, 0.79)], atrial fibrillation [*P* < 0.01, mean rank 1.25 vs. 1.05; bootstrap mean rank difference = 0.20 (95% CI 0.10, 0.31)], and ST-elevation myocardial infarction [*P* = 0.049, mean rank 2.75 vs. 2.02; bootstrap mean rank difference = 0.73 (95% CI 0.31, 1.2)].

Female patients were diagnosed more accurately than males for 6% of sampled cases (3/50). This included two cases of pulmonary embolism (*P* < 1.0 × 10^−9^, mean rank: 1.42 vs. 1.98; bootstrap mean rank difference = 0.56 [95% CI: 0.41, 0.71], *P* < 0.01, mean rank: 10.04 vs. 10.52; bootstrap mean rank difference = 0.48 [95% CI: 0.12, 0.84]) and deep vein thrombosis (*P* < 1.0 × 10^−5^, mean rank: 1.02 vs. 1.43; bootstrap mean rank difference = 0.41 [95% CI: 0.26, 0.58]).

### Anxiety misdiagnosis

Of the 11 cases with significant differences in diagnostic accuracy by gender, GPT-4o more often attributed female patients’ symptoms to anxiety or panic disorder compared to males in 4 cases (36%; *Figure 2D*). Female patients were more often misdiagnosed with anxiety instead of atrial fibrillation (*P* < 1.0 × 10^−5^, mean rank 6.48 vs. 8.06; bootstrap mean rank difference = 1.58 [95% CI: 1.0, 2.2]), cardiomyopathy [*P* < 0.001, mean rank: 9.13 vs. 10.18; bootstrap mean rank difference = 1.05 (95% CI: 0.59, 1.5)], non-ST-elevation myocardial infarction [*P* = 0.014, mean rank: 9.94 vs. 10.39; bootstrap mean rank difference = 0.45 (95% CI 0.15, 0.76)], and atrial flutter [*P* = 0.047, mean rank: 5.73 vs. 6.38; bootstrap mean rank difference = 0.65 (95% CI: 0.18, 1.1)], indicating that GPT-4o may perpetuate harmful stereotypes about women during clinical reasoning. The remaining seven cases showed no statistically significant differences in the diagnostic rank of anxiety or panic disorders, with four cases not including any anxiety or panic diagnoses across all differential diagnoses.

## Discussion

In the majority of conditions examined, GPT-4o and GPT-4 underrepresented women in simulated cardiovascular cases. Educational materials that are demographically representative and accurate are important for training equitable healthcare practitioners.^11^ Underrepresentation of female patients may contribute to delayed recognition of cardiovascular diseases, a key contributor to worse outcomes.

GPT-4o also showed lower diagnostic accuracy for female patients in 16% of sampled cases, in comparison to 6% of cases with higher accuracy for female patients. While effect sizes were modest, with the largest mean rank difference on the differential only around 2.88, small shifts in diagnostic ranking can affect early testing in time-critical presentations. Nonetheless, our study does not quantify delays, and the clinical significance of these differences remains to be established.

Of note, GPT-4o more frequently attributed female patients’ cardiovascular symptoms to anxiety or panic disorder. Such misdiagnosis is well documented in clinical practice, where women’s less ‘classic’ presentations can be mistaken for psychosomatic causes, delaying appropriate care.^10^ In our analysis, GPT-4o ranked anxiety or panic disorders higher in the differential for female patients with atrial fibrillation, cardiomyopathy, non-ST-elevation myocardial infarction, and atrial flutter despite identical patient presentation. These observations raise concern that LLMs could reflect or amplify gendered symptom-attribution patterns.

A potential source of this bias is training data that encodes historical patterns,^5^ linking women’s cardiovascular symptoms with anxiety. For example, if descriptions of female patients’ symptoms frequently co-occur with terms like ‘anxiety,’ ‘stress,’ or ‘palpitations’ in the training data, the model may rank psychiatric causes higher, even if cardiac causes are more likely. This may risk embedding and normalizing diagnostic bias within automated reasoning systems.

Existing literature evaluating GPT, such as that by Zack *et al*.,^6^ has not found consistent patterns of diagnostic accuracy across gender groups. Unlike Zack *et al*., who examined GPT-4 bias broadly using standardized case vignettes, our study adds novelty by focusing on cardiovascular care, using authentic patient documentation that captures real-world complexity, and employing GPT-4o, a newer model with enhanced reasoning capabilities and transparency. Our analysis, therefore, reveals a potential cause for concern for diagnostic bias against women in cardiovascular care, specifically.

These results should be considered hypothesis-generating and context-specific. Limitations to our study include the small sample size of patient notes derived from a single high-volume centre database. Additionally, GPT-4o and GPT-4 were selected due to their popularity and rapid integration into healthcare systems; however, further examination of other LLMs is necessary to evaluate similar biases before their deployment. Moreover, the study’s narrow gender categories only reflect those traditionally used and with available US prevalence data, failing to account for intersectional gender identities.

In summary, these findings suggest that LLM-generated educational materials and diagnostic support could contribute to perpetuating historical gender biases in cardiovascular care if adopted uncritically. While it is unlikely that care providers will solely rely on GPT for educational purposes and diagnostic decisions, biased responses could amplify disparities through automation bias, the tendency to uncritically rely on recommendations,^7^ especially when recommendations may confirm a provider’s own unrecognized biases.

To reduce the likelihood of gender bias in future models, algorithms should incorporate balanced and demographically representative training data, transparent reporting of demographic distributions, and continuous auditing for bias during model updates. Additionally, bias-mitigation strategies, such as counterfactual data augmentation, adversarial debiasing, and prompt-level guardrails, should be prioritized to reduce inequitable outputs. Finally, clinicians already using LLMs should be educated on the risks of automation bias to preserve their independent judgment during clinical decision-making.

## Data Availability

The data underlying this article were accessed from PhysioNet (https://physionet.org/content/mimic-iv-note/, version 2.2). The derived data generated in this research will be shared on reasonable request to the corresponding author with permission from PhysioNet.

## References

[1] Minaee S, Mikolov T, Nikzad N, Chenaghlu M, Socher R, Amatriain X, et al Large Language Models: A Survey. *ArXiv*. 10.48550/arXiv.2402.06196. 9 February 2024, preprint: not peer reviewed.

[2] Fleming SL, Morse K, Kumar A, Chiang C, Patel B, Brunskill E, et al Assessing the Potential of USMLE-Like Exam Questions Generated by GPT-4. *MedRxiv*. 10.1101/2023.04.25.23288588, 2023, preprint: not peer reviewed.

[3] Kanjee Z, Crowe B, Rodman A. Accuracy of a generative artificial intelligence model in a Complex diagnostic challenge. JAMA 2023;330:78–80. 37318797 10.1001/jama.2023.8288 PMC10273128

[4] Quer G, Topol EJ. The potential for large language models to transform cardiovascular medicine. Lancet Digit Health 2024;6:767–771. 10.1016/S2589-7500(24)00151-1 39214760

[5] Kotek H, Dockum R, Sun D. Gender bias and stereotypes in large language models, eds. *Proceedings of the ACM Collective Intelligence Conference, Delft, Netherlands*. New York, United States: Association for Computing Machinery; 2023, p. 12–24.

[6] Zack T, Lehman E, Suzgun M, Rodriguez J, Celi L, Gichoya J, et al Assessing the potential of GPT-4 to perpetuate racial and gender biases in health care: a model evaluation study. Lancet Digit Health 2024;6:12–22. 10.1016/S2589-7500(23)00225-X 38123252

[7] Khera R, Simon MA, Ross JS. Automation bias and assistive AI: risk of harm from AI-driven clinical decision support. JAMA 2023;330:2255–2257. 38112824 10.1001/jama.2023.22557

[8] Weisman D, Sugarman A, Huang YM, Gelberg L, Ganz PA, Comulada WS. Development of a GPT-4-powered virtual simulated patient and communication training platform for medical students to practice discussing abnormal mammogram results with patients: multiphase study. JMIR Form Res 2025;17:e65670. 10.2196/65670 PMC12046251 40246299

[9] Johnson A, Pollard T, Horng S, Celi LA, Mark R. MIMIC-IV-note: deidentified free-text clinical notes (version 2.2). PhysioNet 2023. RRID:SCR_007345. https://physionet.org/content/mimic-iv-note/2.2/

[10] Johnson HM, Gorre CE, Friedrich-Karnik A, Gulati M. Addressing the bias in cardiovascular care: missed & delayed diagnosis of cardiovascular disease in women. Am J Prev Cardiol 2021;30:100299. 10.1016/j.ajpc.2021.100299 PMC8666638 34917995

[11] Turbes S, Krebs E, Axtell S. The hidden curriculum in multicultural medical education: the role of case examples. Acad Med 2002;77:209–216. 11891157 10.1097/00001888-200203000-00007

